# Prolonged intermittent hemodialysis using a standard dialysate flow rate for severe overdose of sustained-release valproic acid: A case report

**DOI:** 10.1016/j.toxrep.2025.102132

**Published:** 2025-09-26

**Authors:** Hiroki Inoue, Satoshi Yoshikawa, Nobuyasu Matsukawa, Atsushi Shima, Takuya Nishizawa, Takeshi Ueda

**Affiliations:** Department of Emergency and General Internal Medicine, Rakuwakai Marutamachi Hospital, 9-7 Jurakumawari-Matsushita-cho, Nakagyo-ku, Kyoto 604-8401, Japan

**Keywords:** Valproic acid, Sustained-release, Overdose, Intermittent hemodialysis, Extracorporeal treatment, Extracorporeal treatments in poisoning (EXTRIP)

## Abstract

**Background:**

Valproic acid poisoning can be life threatening and may require urgent extracorporeal elimination. In particular, sustained-release formulations pose a challenge, as conventional short-duration intermittent hemodialysis may fail to remove the drug sufficiently because of delayed and prolonged drug absorption. While prolonged intermittent hemodialysis is a rational alternative, its clinical effectiveness and safety in cases of severe sustained-release sodium valproate overdose have not been reported.

**Case:**

A woman in her 20 s developed coma after ingesting 45 g (approximately 1100 mg/kg) of sustained-release VPA. Prolonged IHD was delivered for 22 h using a blood flow rate (Qb) of 180 mL/min and a dialysate flow rate (Qd) of 500 mL/min. Her consciousness improved in parallel with a marked decline in serum VPA levels, and she was discharged without any neurological sequelae. A two-point, on-dialysis apparent elimination half-life was estimated to be approximately 2.35 h.

**Conclusion:**

In this case, prolonged IHD appeared to have an effect in decreasing VPA concentrations and was safely implemented with monitoring.

## Highlights

• Severe sustained‑release valproic acid overdose treated with a single 22‑hour session of intermittent hemodialysis.
• The operational parameters: blood flow 180 mL/min; dialysate flow 500 mL/min; triacetate dialyzer 2.1 m².
• Serum VPA decreased rapidly with clinical recovery; safety maintained via serial labs and tailored electrolyte replacement.
• The two-point on-dialysis apparent elimination half-life was 2.35 h (two concentrations measured 6 h apart).

## Graphical Abstract

**Figure:**
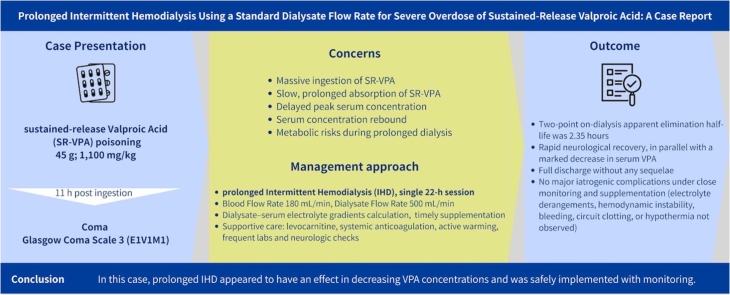

## 1 Introduction

The management of valproic acid (VPA) poisoning is primarily supportive; however, these measures are often inadequate in severe cases of poisoning. Because adjunctive therapies do not directly remove toxins, extracorporeal elimination may be required in life-threatening poisonings. VPA is a suitable candidate for extracorporeal removal because of its low molecular weight (144 g/mol) and small volume of distribution (0.1–0.4 L/kg) [3]. While approximately 90 % of VPA is protein-bound within the therapeutic range [4], protein binding becomes saturated in cases of overdose. This leads to a significant increase in the free drug fraction, reducing protein binding to as low as 15 % [5]. Given these pharmacokinetics, the Extracorporeal Treatments in Poisoning (EXTRIP) workgroup recommends hemodialysis (HD) for high-concentration VPA poisoning [6]. Therapeutic VPA concentrations are 50–100 mg/L; the EXTRIP workgroup uses this range as a criterion for the cessation of extracorporeal treatment in VPA poisoning [6]. Immediate-release formulations typically reach peak serum levels within 7.4 h, whereas sustained-release formulations can take up to 17 h [2], [7]. Due to this delayed absorption, a single 4–6-hour session of intermittent hemodialysis (IHD) may be insufficient. Because this consideration primarily applies when IHD is initiated early after ingestion while absorption from a sustained-release formulation is ongoing, the adequacy of a single 4–6-hour session depends on timing and decontamination. Moreover, if IHD is performed on consecutive days, serum VPA levels may rebound during off-dialysis intervals. Although continuous kidney replacement therapy (CKRT) provides longer-duration clearance, its efficacy is markedly lower than that of IHD [6], [8]. Therefore, prolonged IHD is a rational alternative that combines relatively high clearance with an extended duration. Published clinical data specifically evaluating prolonged IHD in sustained-release sodium valproate (SR-VPA) overdose are limited. This case report describes the clinical course and practical considerations of prolonged IHD in cases of SR-VPA poisoning.

## 2 Case presentation

**Fig. 1:**
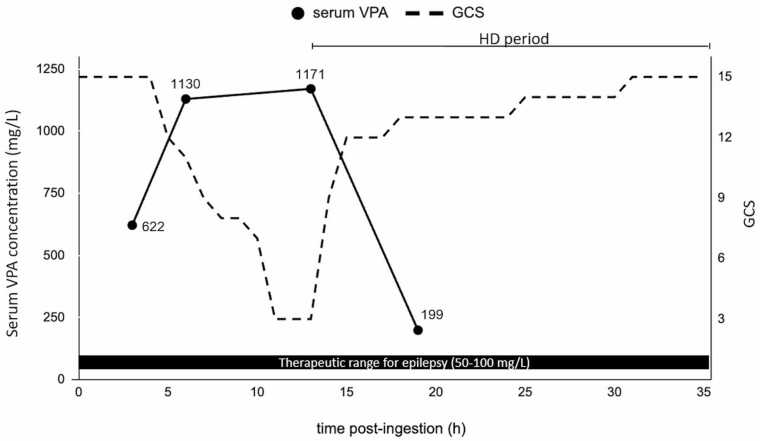
Time course of the serum VPA concentration and GCS. Serum VPA concentration (solid line, left y-axis) and GCS (dashed line, right y-axis) over time post-ingestion. The HD period is indicated in the graph. Therapeutic range for epilepsy (50–100 mg/L) is shown as a black bar.

A woman in her 20 s with a medical history of symptomatic epilepsy had been prescribed SR-VPA (Depakene® R) 400 mg/day and lamotrigine 25 mg/day but had been non-compliant with her medications for several years. She ingested a large overdose of SR-VPA approximately two and a half hours before calling emergency services and was subsequently transported to our hospital. On arrival, her vital signs were as follows: temperature, 37.5°C; blood pressure, 132/82 mmHg; pulse, 75 beats/min; respiratory rate, 20 breaths/min; and SpO₂, 99 % on room air. Her Glasgow Coma Scale (GCS) score was E4V5M6 [GCS 15]. Physical examination revealed no ataxia or tremors. She reported the ingestion of 45 g (approximately 1100 mg/kg) of SR-VPA, which was consistent with the empty blister pack found at the scene. Initial laboratory tests showed normal renal function, serum electrolytes, complete blood count, coagulation profile, blood ammonia levels, and arterial blood gas levels. Gastric lavage was performed via an 18 Fr nasogastric tube while the patient was awake until the aspirate ran clear with no visible pill fragments, followed by a single 50 g dose of activated charcoal. Although she initially remained conscious and mobile, her mental status gradually deteriorated. Brain computed tomography (CT) performed after deterioration showed no signs of cerebral edema. At 11 h post-ingestion, the patient became unresponsive to painful stimuli and lost both oculocephalic and pupillary light reflexes (E1V1M1 [GCS 3]). Despite stable vital signs, profound neurological decline necessitated urgent extracorporeal toxin removal. Indications for initiating IHD were deep coma with loss of brainstem reflexes after massive sustained-release ingestion, with high serum concentrations anticipated. Intermittent hemodialysis (IHD) was initiated along with levocarnitine therapy (1000 mg every 8 h). The dialysis parameters were as follows: Dialysate: Kindaly Dialysis Solution AF-5® (Na⁺ 140 mEq/L, K⁺ 2.3 mEq/L, Ca²⁺ 2.6 mEq/L, Mg²⁺ 1.2 mEq/L, Cl⁻ 113.9 mEq/L, CH₃COO⁻ 4.2 mEq/L, HCO₃⁻ 30 mEq/L, Glucose 150 mg/dL), Membrane: FB-210Pβeco® (Triacetate hollow-fiber dialyzer, surface area 2.1 m²), Vascular Access: Right internal jugular vein via double-lumen catheter, Anticoagulant: Heparin sodium 500 IU/h, Blood flow rate (Qb): 180 mL/min, Dialysate flow rate (Qd): 500 mL/min. After initiation, she gradually regained her response to painful stimuli, and her oculocephalic reflex returned to normal. Her consciousness gradually improved. After 22 h of IHD (35 h post-ingestion), the patient regained full alertness (E4V5M6 [GCS 15]), and IHD was discontinued. Cessation was based on full neurologic recovery to GCS 15 with stable vital signs and laboratory values under ongoing monitoring. Because VPA assays were not available in real time and the patient had regained full alertness while prolonged IHD was ongoing, no further VPA serum testing was performed. Her clinical course is summarized in Table 1, and the time course of serum VPA concentration is shown in Fig. 1, which is inversely correlated with her GCS score. Blood ammonia levels and liver and kidney function test results remained within the institutional reference ranges throughout the admission period. The full values with sampling times are provided in Supplementary Table S1. On hospital day 5, the patient was fully ambulatory and was discharged. At the three-week follow-up, she had returned to her baseline level of functioning without any neurological sequelae and was regularly attending outpatient psychiatric care appointments.

**Table 1:** Clinical course, GCS, and serum VPA concentrations.

Time post-ingestion (h)	Clinical Events and Patient Status	GCS	Serum VPA Concentrations (mg/L)
3	Presentation to hospital; gastric lavage and activated charcoal administered.	E4V5M6	621.5
4	Admission to the High Care Unit (HCU).	E4V5M6	Not measured
5	The patient became agitated and developed tachycardia.	E3V4M5	Not measured
6	While speech production is intact, functional communication is impaired.	E2V4M5	1130
10	The brain CT revealed no evidence of cerebral edema.	E2V1M4	Not measured
11	Oculocephalic reflex became absent.	E1V1M1	Not measured
13	HD was initiated.	E1V1M1	1171
14	Oculocephalic reflex returned.	E2V3M4	Not measured
15	Able to follow motor commands (e.g., hand grasp and release).	E2V4M6	Not measured
19	Patient was somnolent but followed commands.	E3V4M6	199.4
35	Consciousness became clear; HD was discontinued.	E4V5M6	Not measured

This table outlines the patient's clinical course, including key events, GCS scores, and serum VPA concentrations at specific time points following ingestion.

Note: Serum VPA concentrations were not available on the day of admission; the results shown were reported later.

Concentration–time profile and summary of pharmacokinetic approximations. Serum VPA samples were venous specimens analyzed by an external laboratory in daytime batches; real-time testing was unavailable at our facility. Two on-dialysis serum VPA concentrations were available 6 h apart (from 1171 to 199 mg/L) during a single prolonged IHD session. The apparent on-dialysis elimination rate constant (k) was estimated from these two concentrations measured Δt = 6 h apart using a standard one-compartment relation: k = [ln C1 − ln C2]/Δt. This two-point approximation yielded an apparent elimination half-life of approximately 2.35 h. Given the limited sampling, this two-point estimate reflects total elimination (endogenous plus extracorporeal) over the measured interval; apportionment to extracorporeal clearance, and assessment of post-IHD rebound were not possible.

## 3 Discussion

In this case of severe sustained-release valproic acid (SR-VPA) overdose, a single prolonged session of intermittent hemodialysis (IHD) was associated with neurological recovery without major complications. Two pragmatic clinical points follow: first, prolonged IHD may be considered in severe SR-VPA poisoning; second, careful operational monitoring can support its safe implementation.

The patient ingested approximately 1100 mg/kg of SR-VPA, well above the 400 mg/kg toxicity threshold associated with organ failure [1]. After 6 h of IHD, the Glasgow Coma Scale had improved to 13, while the concomitant serum VPA concentration remained above the therapeutic range. Although concentration alone does not determine severity, published data suggest that moderate toxicity generally occurs at concentrations greater than 450 mg/L and severe toxicity (e.g., coma, respiratory depression, metabolic acidosis, shock) at greater than 850 mg/L [2]. In our case, coma (E1V1M1 [GCS 3]) preceded IHD, with recovery to E4V5M6 [GCS 15] during a single 22-h session—directionally consistent with these concentration–effect relationships. Sustained-release formulations can exhibit delayed absorption with secondary peaks, and rebound after early discontinuation has been described. We did not assess rebound in this patient and therefore present this as a general consideration from prior reports rather than as a finding in our case [7], [9]. Thus, a single extended session may be preferable to multiple short sessions in selected severe cases, though the optimal duration remains uncertain and requires further study.

## 4 Toxicokinetics

Using two intradialysis serum VPA concentrations 6 h apart, we derived a two-point, one-compartment apparent elimination half-life of approximately 2.35 h. Details of the calculation are provided in the Case Presentation section. Given that this two-point method reflects total elimination over the interval (endogenous plus extracorporeal) and sampling was limited, we did not estimate the clearance. Delayed absorption from a sustained-release formulation and single-dose activated charcoal may have contributed to the observed decline, and post-IHD rebound was not assessed; therefore, the interpretation is conservative.

## 5 Modality selection and duration

When hemodynamics permits, IHD provides higher solute clearance than continuous kidney replacement therapy (CKRT); the EXTRIP workgroup reported VPA clearances for IHD of 51–140 mL/min versus 1.8–23.9 mL/min for CKRT [6], [10]. In unstable patients, a pragmatic approach is to attempt IHD with vasopressor support and proceed to CKRT if not tolerated. Duration should be individualized according to ingested dose, clinical course, and ideally serial VPA levels; decontamination (e.g., charcoal) can further shape the concentration–time profile.

## 6 Treatment duration and timing

In this case, most of the clinical improvement and the largest decline in serum VPA levels occurred during the first 6 h of IHD. We acknowledge that in selected scenarios, shorter IHD sessions can be sufficient, particularly when therapy is initiated later relative to ingestion and/or when gastrointestinal decontamination is effective. However, with sustained-release formulations, ongoing absorption and delayed peaks can yield rebound after early discontinuation, as described in previous reports [9]. In our patient, IHD began approximately 11 h after ingestion, within the window in which sustained-release preparations may still be absorbed. Balancing these considerations, we elected to continue a single prolonged session, while recognizing that a shorter treatment might have sufficed; therefore, the optimal duration should be individualized according to timing, decontamination, hemodynamics, and—where available—serial VPA measurements.

One of the main concerns with prolonged IHD is the risk of iatrogenic complications, such as electrolyte imbalances (e.g., hypokalemia, hypomagnesemia, hypophosphatemia, and hypercalcemia) and metabolic alkalosis, due to excessive solute removal. We mitigated these via gradient-aware prescriptions, frequent laboratory monitoring, and timely replacement. For example, with Qb = 180 mL/min and hematocrit 40 %, the plasma flow is 108 mL/min (6.48 L/h); with serum K 3.2 mEq/L and dialysate K 2.3 mEq/L, assuming near-equilibration, approximately 5.8 mEq/h of K would be removed, so approximately 6 mEq/h was supplemented, with adjustments made as necessary based on the measured values. Maintaining plasma tonicity is crucial in VPA overdose, where cerebral edema can occur as a complication. Effective plasma tonicity (2 × [Na⁺] + [blood glucose]/18) was monitored to avoid abrupt osmotic shift. Using the same diffusion framework as that for potassium, with serum sodium 143 mEq/L and dialysate sodium 140 mEq/L, the predicted net diffusive sodium loss was approximately 19.4 mEq/h ([143 −140] × 6.48). Although this yielded a theoretical replacement requirement of 19.4 mEq/h, we deliberately adopted a conservative strategy and initiated replacement below the estimate (3 % sodium chloride at 20 mL/h [0.513 mEq/mL; approximately 10.3 mEq/h]) with an a priori plan to titrate upward if serum sodium or effective plasma tonicity declined. Serum sodium and effective plasma tonicity remained stable; therefore, this rate was continued for the duration of IHD. Detailed trajectories and dialysate compositions are provided in Supplementary Table S2A, and dosing frequency and cumulative amounts are provided in Supplementary Table S2B. Early extracorporeal removal may help correct acidemia when it is present. In this case, prolonged IHD was performed without circuit clotting or significant bleeding despite heparin use, and hypothermia was prevented with external warming.

## 7 Limitations

A key limitation of this report was our facility's inability to measure serum VPA concentrations in real time. During the ongoing 22 h IHD session, the patient’s neurologic status normalized, and additional sampling was not anticipated to alter management; therefore, no further VPA serum concentrations were obtained. The limited sampling precludes precise clearance estimation and assessment of post-IHD rebound; where feasible, rapid assays could support de-escalation strategies (e.g., transitioning from IHD to CKRT) and more individualized management [11]. Overall, in the absence of established guidelines, treatment duration should be individualized based on serial clinical assessments and, when available, serum VPA measurements; adjusting Qd or transitioning to lower-efficiency modalities may offer flexibility while minimizing adverse effects.

## 8 Conclusion

In this severe SR-VPA overdose, prolonged IHD was associated with clinical improvement and declining VPA concentrations, and it was implemented safely under close monitoring. Given the limited sampling, our interpretation is conservative, and further work is needed to define optimal practice.

## CRediT authorship contribution statement

**Hiroki Inoue:** Conceptualization, Data curation, Investigation, Writing – original draft, Writing – review & editing. **Satoshi Yoshikawa:** Conceptualization, Investigation. **Nobuyasu Matsukawa:** Conceptualization, Investigation. **Atsushi Shima:** Conceptualization, Investigation. **Takuya Nishizawa:** Conceptualization, Investigation. **Takeshi Ueda:** Supervision.

## Ethics statement

This study was conducted in accordance with the principles of the Declaration of Helsinki. Written informed consent for the publication of this report was obtained from the patient, and all identifiable information was anonymized.

## Funding

This research did not receive any specific grants from funding agencies in the public, commercial, or not-for-profit sectors.

## Declaration of Generative AI and AI-assisted technologies in the writing process

During the preparation of this work, the author used Gemini 2.5 Pro and Paperpal to improve the language. After using these tools/services, the author reviewed and edited the content as needed and took full responsibility for the content of the publication.

## Declaration of Competing Interest

The authors declare that they have no known competing financial interests or personal relationships that could have appeared to influence the work reported in this paper.

## Data Availability

No data was used for the research described in the article.
