# Supplementary material for: Prolonged intermittent hemodialysis using a standard dialysate flow rate for severe overdose of sustained-release valproic acid: A case report
Source: Toxicol Rep. 2025 Sep 26;15:102132. doi: 10.1016/j.toxrep.2025.102132 (PMC12513118; doi:10.1016/j.toxrep.2025.102132)
Supplement: Supplementary file 1 — Supplementary material [file mmc1.docx]

Supplementary Table S1. Serial ammonia, liver, and kidney function tests with sampling times, units, and reference ranges.

| Time post ingestion (h) | 3 | 6 | 13 | 19 | 35 |
| --- | --- | --- | --- | --- | --- |
| Time after IHD initiation (h) |  |  | 0 | 6 | 22 |
| Serum VPA (mg/L) | 622 | 1130 | 1171 | 199 | N/A |
| Ammonia (μmol/L) , Ref: ≦ 45 | 28.8 | 19.4 | N/A | 26.4 | N/A |
| ALT (U/L) | 10 | 8 | N/A | N/A | 12 |
| ALP (U/L) | 79 | 77 | N/A | N/A | 61 |
| LDH (U/L) | 134 | 127 | N/A | N/A | 162 |
| Urea nitrogen (mmol/L) | 3.14 | 2.93 | 2.18 | N/A | 0.39 |
| Creatinine (mg/dL) | 0.7 | 0.78 | 0.42 | N/A | 0.33 |

Ref = institutional reference interval. Values are in SI units.

Supplementary Table S2A. Changes in laboratory parameters following initiation of IHD and dialysate electrolyte composition

| Time after IHD initiation (h) | 0 | 8 | 22 | Dialysate Electrolyte Composition |
| --- | --- | --- | --- | --- |
| Sodium (mEq/L) | 143 | 142 | 144 | 140 |
| Potassium (mEq/L) | 3.2 | 3.3 | 3.8 | 2.3 |
| Chloride (mEq/L) | 104 | 106 | 109 | 114 |
| Phosphate (mg/dL) | 1.7 | 3.2 | 2.8 | 0 |
| Magnesium (mg/dL) | 2.1 | 2.6 | 2.4 | 1.2 |
| ionized Ca (mmol/L) | 0.92 | 1.07 | 1.09 | 1.3 |
| Glucose (mmol/dL) | 6.56 | 5.59 | 5.94 | 5.56 |
| Urea nitrogen (mmol/L) | 2.18 | N/A | 0.39 | 0 |
| pH | 7.426 | 7.331 | 7.391 |  |
| Bicarbonate (mmol/L) | 19.6 | 22.6 | 24.0 | 30 |
| Tonicity (mOsm/kgH2O) | 295 | 290 | 294 | 290 |

Supplementary Table S2B. Supplementation during prolonged IHD

| Time window | IHD hour 0–22 (11–35 h post-ingestion) |
| --- | --- |
| Modality details | Qb^†^ 180 mL/min; Qd^‡^ 500 mL/min; triacetate 2.1 m² |
| Serum Na (mEq/L) | 143 |
| Dialysate Na (mEq/L) | 140 |
| Estimated net Na flux* (mEq/h) | approximately 19.4 |
| Sodium supplementation (solution; rate) | 3% NaCl IV, 20 mL/h (0.513 mEq/mL ≈ 10.3 mEq/h) |
| Cumulative dose (mEq) | 226.6 |

* Net diffusive sodium flux was estimated as (Serum Na−Dialysate Na) × plasma flow; plasma flow was estimated as Qb × (1−hematocrit) =180 × 0.60=108 mL/min (approximately 6.48 L/h). This simplified calculation assumes near-equilibration. Effective plasma tonicity = 2 × [Na⁺] + [blood glucose]/18. 3% sodium chloride concentration = 0.513 mEq/mL. † Qb; blood flow rate (mL/min), ‡ Qd; dialysate flow rate (mL/min)
